# Harnessing *Toxoplasma gondii* microneme proteins for serodiagnosis and vaccine development: a review

**DOI:** 10.1080/07853890.2026.2667678

**Published:** 2026-05-11

**Authors:** Yanfei Ji, Zhihao Wang, Yuxuan Feng, Irfan Ahmad, Khalid J. Alzahrani, Khalaf F. Alsharif, Fuad M. Alzahrani, Xia Zhao, Abdul Qadeer, Wenqiang Liu

**Affiliations:** a College of Agriculture and Biology, Liaocheng University, Liaocheng, China; b Northeast Agricultural University College of Veterinary Medicine, Harbin, China; c Department of Clinical Laboratory Sciences, College of Applied Medical Sciences, King Khalid University, Abha, Saudi Arabia; d Department of Clinical Laboratories Sciences, College of Applied Medical Sciences, Taif University, Taif, Saudi Arabia; e School of Medical Sciences, Shandong Xiehe University, Jinan, P. R. China

**Keywords:** *Toxoplasma gondii*, microneme proteins, host cell invasion, immunomodulation, serodiagnosis, vaccine development

## Abstract

**Introduction:**

*Toxoplasma gondii* (*T. gondii*), an obligate intracellular apicomplexan parasite, infects virtually all warm-blooded animals through a sophisticated sequential invasion mechanism involving coordinated secretion from specialized apical organelles. Among these secretory proteins, microneme proteins (MICs) serve as central molecular effectors orchestrating host-parasite interactions. This review comprehensively examines the structural organization, regulatory mechanisms, and diverse functions of MICs in *T. gondii* pathogenesis, and evaluates their translational potential for diagnostics and vaccine development.

**Discussion:**

Key MIC adhesin complexes – including the lectin-based MIC1/4/6 complex and the transmembrane MIC2-M2AP complex – mediate glycan recognition, host cell attachment, moving junction formation, and gliding motility. Beyond invasion, MICs function as immunomodulators engaging Toll-like receptors (TLR2, TLR4, TLR11) to elicit pro-inflammatory responses while activating EGFR-Akt signaling to inhibit autophagy. The translational potential is substantial: MIC-based antigens enable sensitive serodiagnosis, differentiating acute from chronic infections. In contrast, MIC-based vaccines across multiple platforms demonstrate considerable protective efficacy in preclinical experimental models. Notably, the MIC1-3 knockout strain confers protection in sheep that is comparable to that of commercial vaccines; however, it is important to note that most supporting evidence remains at the preclinical stage and clinical validation in human populations is still required.

**Conclusions:**

MICs represent promising targets that bridge fundamental parasitology and clinical applications. Integration of MIC biology with translational strategies provides foundations for developing next-generation diagnostics and vaccines against toxoplasmosis.

## 1. Introduction

*Toxoplasma gondii* (*T. gondii*) is an obligate intracellular protozoan parasite belonging to the phylum Apicomplexa [1], which includes other medically important pathogens such as *Plasmodium* species (causative agents of malaria), *Cryptosporidium*, and *Eimeria* [2–4]. This ubiquitous parasite infects virtually all warm-blooded animals, including humans, and is responsible for toxoplasmosis, a disease of significant public health concern [5–7]. It is estimated that over three-fifths of some populations are infected with *T. gondii*, with seroprevalence varying considerably among different geographic regions and increasing with age [8–10].

**Figure 1.**
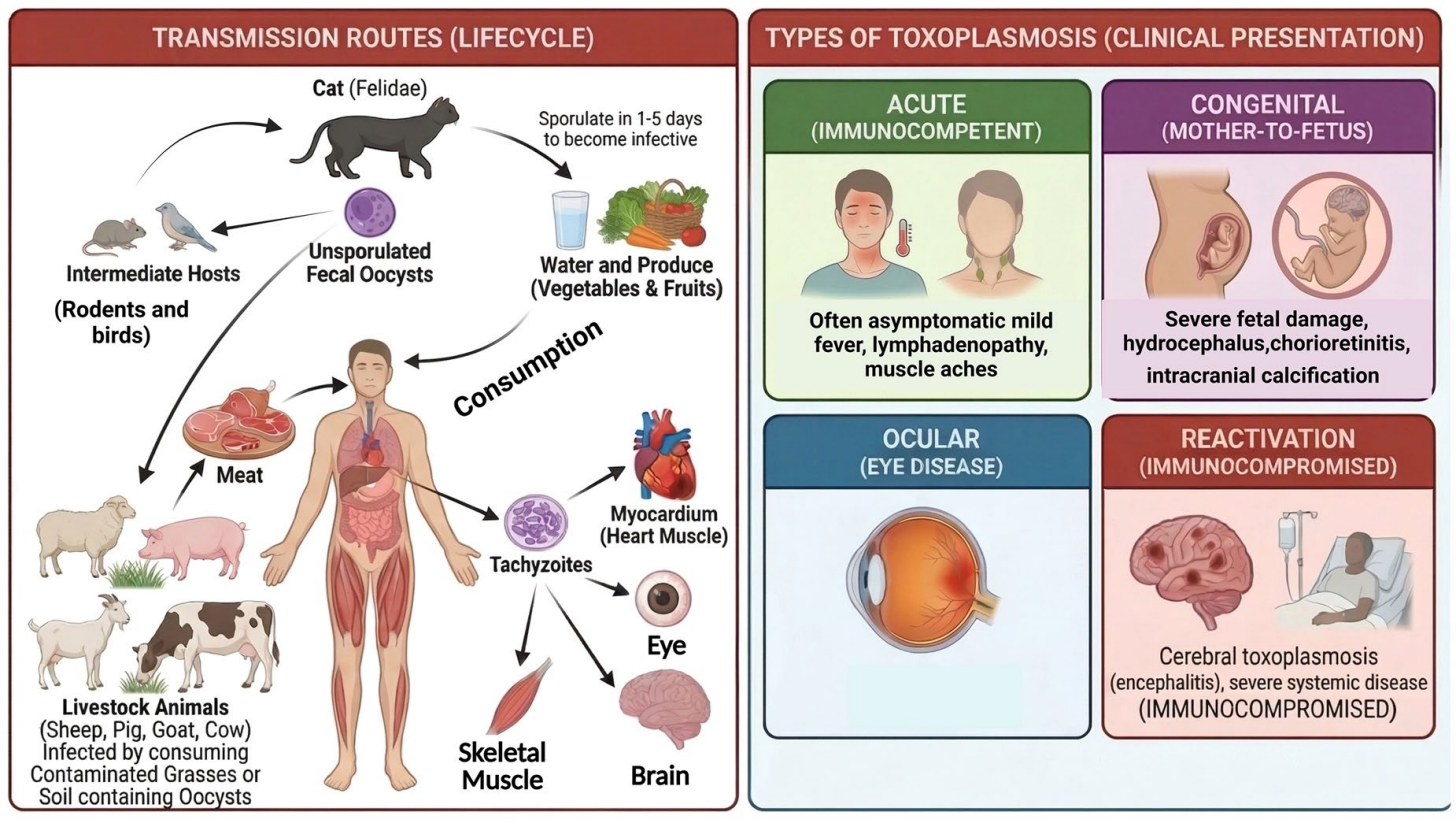
Life cycle, transmission routes, and clinical manifestations of *Toxoplasma gondii* infection. (Left panel) Transmission routes and life cycle: Cats (Felidae) serve as the definitive hosts, shedding unsporulated fecal oocysts that sporulate within 1–5 days in the environment, becoming infective. Intermediate hosts (rodents, birds) and livestock (sheep, pig, goat, cow) – acquire infection by ingesting oocysts from contaminated grasses or soil, and develop tissue cysts. Humans become infected *via* three primary routes: (1) consumption of undercooked meat from infected animals containing tissue cysts; (2) ingestion of oocyst-contaminated water, vegetables, or fruits; and (3) congenital (vertical) transmission. Once inside the human host, tachyzoites disseminate hematogenously to target organs, including the brain, eye, skeletal muscle, and myocardium (heart muscle). (Right panel) Clinical presentations of toxoplasmosis: Acute toxoplasmosis in immunocompetent individuals is often asymptomatic or presents with mild flu-like symptoms, including low-grade fever, lymphadenopathy, and muscle aches. Congenital toxoplasmosis results from mother-to-fetus vertical transmission and can cause severe fetal damage, including hydrocephalus, chorioretinitis, and intracranial calcifications. Ocular toxoplasmosis manifests as ocular disease (eye disease) with retinochoroiditis and potential vision impairment. Reactivation of toxoplasmosis occurs in immunocompromised patients and can lead to life-threatening cerebral toxoplasmosis (encephalitis) and severe systemic disease.

The complex life cycle of *T. gondii* involves both definitive and intermediate hosts, with multiple transmission routes contributing to its widespread distribution (Figure 1). Felids, particularly domestic cats, serve as the definitive hosts of sexual reproduction, resulting in the shedding of environmentally resistant oocysts in feces [11]. These oocysts sporulate in the environment and can contaminate water and food sources, leading to infection of intermediate hosts, including rodents, birds, livestock, and humans [12]. Human infection primarily occurs through three main routes: ingestion of tissue cysts in undercooked or raw meat from infected livestock, consumption of food or water contaminated with sporulated oocysts, and vertical transmission from mother to fetus during primary infection (Figure 1) [9,10]. Additionally, rare cases of transmission through blood transfusion and organ transplantation have been documented [13,14].

The clinical manifestations of toxoplasmosis vary significantly depending on the host’s immune status and the timing of infection (Figure 1). While primary infection in immunocompetent individuals is typically asymptomatic or presents as mild, self-limiting lymphadenopathy, toxoplasmosis poses severe risks to specific populations [15,16]. Congenital toxoplasmosis, resulting from vertical transmission during primary maternal infection, can cause devastating consequences, including spontaneous abortion, hydrocephalus, intracranial calcifications, and chorioretinitis – the classic triad of congenital disease [17,18]. In immunocompromised individuals, particularly those with AIDS or organ transplant recipients, reactivation of latent infection can lead to life-threatening encephalitis [16,19]. Additionally, ocular toxoplasmosis represents a leading cause of posterior uveitis worldwide [20,21].

The remarkable success of *T. gondii* in establishing infection across such a broad host range is attributed to its sophisticated invasion machinery [22]. Unlike many other intracellular pathogens that rely on host cell phagocytosis, apicomplexan parasites actively invade host cells using a unique mechanism driven by a complex interplay between secretory organelles and an actin-myosin motor system [23,24]. *T. gondii* possesses endoplasmic reticulum (ER) and Golgi apparatus, as well as specialized secretory organelles, including micronemes, rhoptries, and dense granules [25]. Upon invasion of host cells, these organelles secrete proteins with various functions to assist tachyzoites in entering host cells and forming a parasitophorous vacuole (PV) to replicate by binary division [26].

During invasion, *T. gondii* first recognizes host cells through surface antigen proteins (SAGs) [27,28], which allow the apical secretory organelles of *T. gondii* to adhere to the host cell surface. Subsequently, micronemes secrete microneme proteins (MICs), which promote mutual recognition between *T. gondii* and the host surface-related receptors [29,30], identify suitable invasion sites to advance parasite invasion, and assist in the formation of the moving junction (MJ) structure [31]. The rhoptry organelles then secrete rhoptry proteins (ROPs) and rhoptry neck proteins (RONs) [32]. RONs contribute to the formation of the MJ structure, which promotes *T. gondii* invasion of host cells. ROPs can manipulate host cell pathways, including those that control innate immunity and are involved in PV formation, thus contributing to invasion and proliferation [33]. After *T. gondii* enters host cells, dense granule proteins (GRAs) promote PV formation, which is critical for proliferation [34]. These proteins, which are virulence factors of the parasite, all require ER and Golgi trafficking to the secretory organelles [35]. MIC, ROPs/RONs, and GRAs all depend on their N-terminal domain signal peptides to be transported from the ER-Golgi system or trans-Golgi network to the corresponding organelles in soluble or aggregated complexes [36,37] (Figure 1).

**Figure 2.**
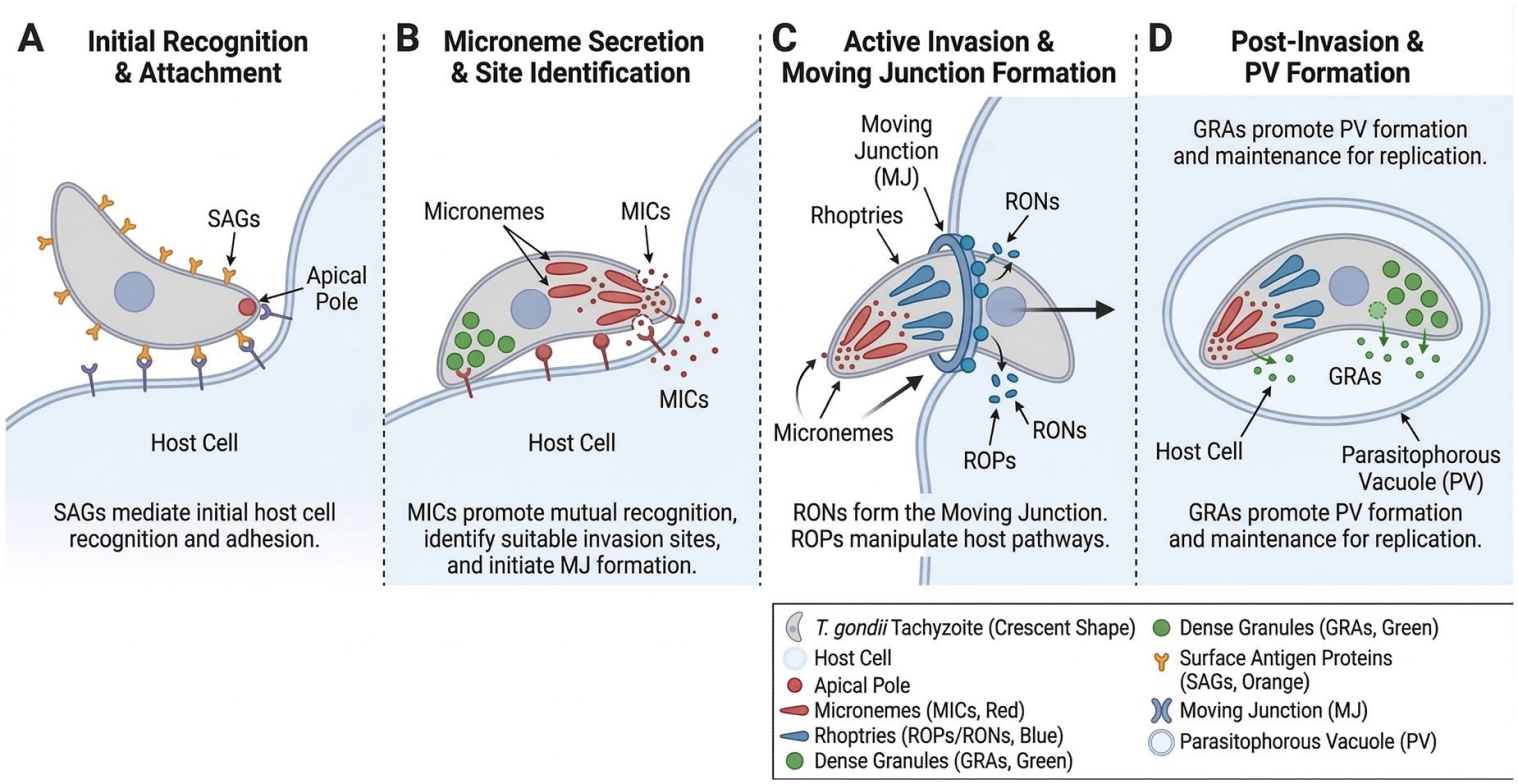
Sequential invasion mechanism of *Toxoplasma gondii* driven by apical secretory organelles. (A) Initial Recognition and Attachment: Surface antigen proteins (SAGs, orange) on the tachyzoite surface mediate initial host cell recognition and adhesion, allowing the apical pole containing secretory organelles to orient toward the host cell membrane. (B) Microneme Secretion and Site Identification: Micronemes (red) discharge microneme proteins (MICs) that promote mutual recognition between the parasite and host cell surface receptors, identify suitable invasion sites, and initiate moving junction (MJ) formation. (C) Active Invasion and Moving Junction Formation: Rhoptries (blue) secrete rhoptry neck proteins (RONs) and rhoptry proteins (ROPs). RONs integrate into the host cell membrane to form the moving junction (MJ, yellow ring), a ring-like structure that the parasite uses to propel itself into the host cell. ROPs are injected into the host cytoplasm to manipulate host cell signaling pathways and facilitate the formation of parasitophorous vacuoles (PVs). (D) Post-Invasion and PV Formation: Following complete entry, dense granules (green) release dense granule proteins (GRAs) that modify and maintain the parasitophorous vacuole (PV), creating a replication-competent compartment that protects the parasite from host cell defense mechanisms while enabling nutrient acquisition and intracellular replication. The *T. gondii* tachyzoite is depicted as a crescent-shaped organism with its characteristic apical pole containing organized secretory organelles: micronemes (red, small, elongated structures), rhoptries (blue, club-shaped organelles), and dense granules (green, spherical organelles distributed throughout the cytoplasm).

Central to the invasion process are the micronemes, specialized secretory organelles located at the apical pole of the parasite that contain adhesive proteins crucial for host cell recognition and attachment [38,39]. Microneme proteins (MICs) constitute a diverse family of molecules that share structural motifs with adhesive domains found in higher eukaryotic proteins, including thrombospondin type 1 repeats (TSR), epidermal growth factor (EGF)-like domains, and integrin-like domains [40–44]. These proteins are released sequentially during invasion, forming complexes that mediate initial attachment to host cells and subsequently facilitate the formation of the moving junction – a ring-like structure through which the parasite propels itself into the host cell (Figure 2) [45–47].

Despite significant advances in our understanding of MIC protein structure and function, several critical gaps remain in the literature. First, while individual MIC proteins have been extensively studied, the relative hierarchy of their contributions to invasion – and the extent of functional redundancy among them – is incompletely understood. Second, translational progress has been hampered by a paucity of head-to-head comparisons of MIC-based diagnostic antigens under standardized conditions, and by the absence of human clinical trial data for any MIC-based vaccine candidate. Third, the impact of strain-specific variation in MIC expression and antigenicity on diagnostic performance and vaccine cross-protection has received limited systematic attention. This review addresses these gaps by providing a critically integrated analysis of MIC protein biology and by evaluating translational evidence considering its current limitations.

This review provides a comprehensive examination of the role of microneme proteins in *T. gondii* pathogenesis, encompassing their molecular architecture, functional properties, regulatory mechanisms, and potential applications in diagnostic and therapeutic interventions against toxoplasmosis.

## 2. Literature search strategy

A comprehensive search of the PubMed/MEDLINE, Scopus, and Web of Science databases was conducted to identify relevant studies published up to March 2025. Search terms included, but were not limited to, the following: ‘*Toxoplasma gondii* microneme proteins’, ‘MIC proteins toxoplasmosis’, ‘*T. gondii* serodiagnosis’, ‘toxoplasmosis vaccine’, ‘MIC1’, ‘MIC2’, ‘MIC3’, ‘MIC4’, ‘MIC6’, ‘host cell invasion apicomplexan’, and ‘toxoplasmosis immunomodulation’. Reference lists of retrieved articles were hand-searched for additional relevant studies. Inclusion criteria encompassed original research articles, systematic reviews, and narrative reviews reporting on MIC protein structure, function, immunology, diagnostic utility, or vaccine efficacy. Studies not in English, conference abstracts without full-text data, and studies reporting exclusively on non-MIC secretory organelle proteins were excluded. No formal PRISMA protocol was applied, as this is a narrative review; however, the search strategy was designed to minimize selection bias and ensure comprehensive coverage of the relevant literature.

## 3. Classification and structural organization of microneme proteins

Microneme (MIC) proteins play important roles in the recognition, adhesion, and invasion of host cells by *T. gondii* [39,48,49]. To date, more than 20 microneme proteins have been identified and characterized in *T. gondii*, each exhibiting distinct structural features and functional properties [50–52]. These proteins can be broadly classified into two categories based on their membrane topology: transmembrane proteins that span the parasite plasma membrane and serve as bridges between host cell receptors and the parasite’s actomyosin system, and soluble proteins that function primarily as adhesins[53,54]. While this binary classification provides a useful organizational framework, it is important to recognize that individual MIC proteins within each category differ substantially in their binding partners, host cell ligands, and downstream signalling roles. Furthermore, many MICs function not as isolated proteins but as interdependent multiprotein complexes, and disruption of any single component can compromise the trafficking and surface presentation of the entire complex. This cooperative architecture has important implications for both diagnostic antigen design and vaccine formulation, as targeting a single protein may be insufficient to neutralize complex-mediated invasion.

### 3.1. The MIC1/4/6 soluble adhesin complex

The microneme proteins MIC1 and MIC4 from *T. gondii* form a crucial adhesin complex, facilitated by the escort protein MIC6, which is exposed on the parasite surface during host cell invasion [40]. These proteins function as lectins, with MIC1 binding terminal sialic acid residues and MIC4 recognizing galactose termini on host cell glycans [55–57]. MIC4 is a 65-kDa protein composed of six apple domains, adhesive modules found in blood coagulation factors and other mammalian proteins [58,59]. MIC4 binds to terminal galactose residues, complementing the sialic acid-binding specificity of MIC1 [56]. MIC6 contains multiple EGF-like domains and serves as the escorter for the complex, ensuring proper sorting to micronemes through its cytoplasmic targeting signals [60,61]. Genetic disruption of MIC1 results in mislocalization of both MIC4 and MIC6, demonstrating the interdependence of complex members for proper trafficking [58].

A structural study reveals the precise molecular basis of TgMIC1’s carbohydrate-binding specificity, demonstrating through crystallography that its micronemal adhesive repeat (MAR) region preferentially binds α2-3-linked sialyl oligosaccharides *via* a unique water-mediated hydrogen-bond network [55]. The research identifies potent fluorinated analogs as high-affinity ligands and provides evidence for an unusual C–F···H–O hydrogen bond within the lectin-carbohydrate complex. These findings define the structural determinants of host cell recognition by TgMIC1 and position it within a newly identified family of sialic-acid binding sites shared by lectins from diverse pathogens [55]. Research has identified TgMIC13 as a novel sialic acid-binding lectin that works alongside TgMIC1 to mediate host cell recognition [56]. It reveals an entire family of microneme adhesive repeat (MAR) domain proteins in coccidian parasites, suggesting a common strategy for broad host range *via* sialylated glycoconjugate targeting [56]. Taken together, the MIC1/4/6 complex exemplifies a hierarchical organizational principle in which a structural escorter (MIC6) is indispensable not only for intracellular trafficking but also for connecting extracellular adhesion to the actin-based motility machinery through its aldolase-binding cytoplasmic tail [62]. The complementary glycan specificities of MIC1 (sialic acid) and MIC4 (galactose) likely expand the parasite’s host range by enabling recognition of multiple terminal sugar motifs on diverse host cell glycoproteins. The discovery of additional MAR-domain lectins such as MIC13 [56] further suggests that sialic acid-targeting is a redundant, evolutionarily reinforced strategy that may limit the efficacy of interventions targeting any single lectin in isolation.

### 3.2. The MIC2-M2AP transmembrane complex

It is well known that *T. gondii* tachyzoites invade host cells by releasing adhesive microneme proteins (MICs) [63]. The MIC2–M2AP complex is abundantly secreted from micronemes, where MIC2 serves as a transmembrane adhesin. The M2AP propeptide is essential for the MIC2–M2AP complex to exit the early endosome, while M2AP cleavage is required for rapid mobilization of the complex from micronemes to the parasite surface. Conditional expression of MIC2 revealed its critical role in directing M2AP to the micronemes. Parasites lacking functional MIC2 were severely impaired in invasion, exhibited predominantly non-productive circular gliding motility, and failed to establish infection in mice at usually lethal doses. These findings highlight the essential role of MIC2 in invasion-related protein trafficking and parasite virulence [64].

Structural studies of the MIC2–M2AP interaction solved the crystal structure of M2AP bound to TSR6 of MIC2, identifying His-620 as critical for binding and demonstrating that residue Y602 is conformationally flexible – locked in the ‘flipped-out’ position upon M2AP engagement, stabilizing the complex [51]. These structural insights provide atomic-level detail that could guide the design of small-molecule inhibitors targeting this interface [51]. M2AP additionally utilizes a modified galectin fold – a structural motif shared with the C-terminal domain of MIC1 – to mediate protein-protein interactions within the complex [65], highlighting an unexpected architectural convergence between the soluble MIC1/4/6 and transmembrane MIC2–M2AP systems. The first crystal structure of the apicomplexan A/I domain of MIC2 reveals a canonical von Willebrand factor A fold with a uniquely configured MIDAS stabilized by the TgMIC2-specific residue Gly185, further distinguishing this adhesin from its mammalian counterparts [66].

It is noteworthy, however, that studies examining the essentiality of MIC2 have produced conflicting conclusions. Whereas conditional knockdown experiments demonstrated severe impairment of invasion and lethality in murine models [64], a subsequent functional analysis found that MIC2 deletion produced only mild virulence attenuation and did not abolish gliding motility, suggesting a modulatory rather than indispensable role [67]. This discrepancy likely reflects differences in experimental design – particularly the distinction between conditional suppression and genetic deletion – and underscores the need for careful interpretation of MIC2-centred therapeutic or diagnostic strategies. Notwithstanding these uncertainties, the atomic-level characterization of the MIC2–M2AP interface [51,66], remains the most promising foundation for structure-guided drug discovery targeting this complex.

## 4. Regulatory mechanisms of microneme secretion

A study elucidates a key signaling mechanism controlling microneme secretion, revealing that the host protein serum albumin acts as a natural trigger by activating a cGMP-dependent protein kinase G (PKG) pathway [68]. This pathway operates independently of elevated intracellular calcium, though calcium can augment the response, demonstrating that PKG activity is the central regulator of MIC protein deployment during invasion. This finding is particularly significant because it demonstrates that the parasite actively senses host-derived soluble factors rather than relying solely on physical contact with host cell surfaces, adding an environmental surveillance dimension to the regulation of MIC secretion [68].

Complementing the PKG pathway, another study delineates a crucial lipid-signaling axis that controls microneme secretion, identifying the balance between diacylglycerol (DAG) and phosphatidic acid (PA) as essential for regulated exocytosis [69]. An apicomplexan-specific DAG-kinase-1 maintains this balance, and an acylized pleckstrin-homology domain protein (APH) on the microneme surface acts as a PA sensor to directly trigger exocytosis [69]. These two regulatory axes – the PKG pathway responding to extracellular host signals and the DAG/PA lipid axis governing membrane fusion at the organelle level – appear to operate in a hierarchical manner, with upstream host cues ultimately converging on lipid-mediated exocytic release. Disrupting either node could therefore impair MIC secretion, making both pathways attractive targets for anti-invasion intervention [68,69].

Host cell entry by the Apicomplexa is associated with the sequential secretion of invasion factors from specialized apical organelles [70]. Secretion of MIC complexes by *T. gondii* facilitates parasite gliding motility, host cell attachment and entry, and egress from infected cells. The shedding of MICs during these steps is mediated by micronemal protein proteases MPP1, MPP2, and MPP3 [70].

### 4.1. Proteolytic processing by rhomboid proteases

Building on the intricate functional roles of MICs in invasion, a study defines the critical post-translational regulation of these adhesins by identifying ROM4 as the primary rhomboid protease responsible for cleaving transmembrane adhesins such as MIC2 [71]. Deletion of ROM4 disrupts the shedding process, causing adhesins to accumulate on the parasite surface, which subsequently leads to non-productive attachment, altered gliding motility, and impaired moving junction formation. Importantly, however, the viability of parasites even in the absence of all three ROMs indicates that robust ROM-independent backup mechanisms for adhesin removal exist [71], pointing to a level of functional redundancy that may complicate therapeutic strategies targeting rhomboid proteases. A complementary study establishes that TgROM4 regulates the surface landscape of multiple adhesins – including MIC2, AMA1, and MIC3 – by maintaining the apical-posterior adhesion gradient required for productive directional motility; parasites with suppressed TgROM4 adhere more but invade less, demonstrating that shedding is critical not merely for adhesin turnover but for spatial polarity [72]. Taken together, these studies establish proteolytic processing as an essential regulatory step that is mechanistically separable from, yet functionally coupled to, adhesin-mediated attachment. The viability of parasites lacking all three ROMs [71], indicates the existence of backup shedding mechanisms whose identity and targetability remain important open questions.

Limited proteolysis of surface proteins accompanies *T. gondii* cell invasion and facilitates parasite migration. Genetic ablation of MIC5 enhances proteolytic processing of several micronemal proteins in tachyzoites – a phenotype reversed by complementation or treatment with the protease inhibitor ALLN, which blocks MPP2 [73]. Despite lacking obvious membrane-association signals, MIC5 localizes to the parasite surface during invasion and its knockout effects extend to non-micronemal secretory proteins such as GRA1, suggesting MIC5 either directly regulates MPP2 activity or modulates substrate accessibility [73]. This positions MIC5 as an indirect regulator of the proteolytic cascade rather than a conventional adhesin, adding a layer of complexity to the regulatory landscape of MIC shedding.

### 4.2. Intracellular trafficking and sorting mechanisms

A study elucidates a fundamental trafficking mechanism shared by microneme proteins (MICs), identifying that short propeptide domains containing conserved aliphatic residues (valine or leucine) at their N-terminal are essential and interchangeable for correct targeting to the microneme organelles [74]. This common pathway ensures the proper localization of diverse MICs, which underpin their multifaceted roles in invasion, immune modulation, and signaling. These findings connect a basic cellular trafficking code to the broader functional repertoire of MIC proteins, ranging from adhesin complexes (e.g. MIC1/4/6, MIC2/M2AP) and immune activation (e.g. MIC3, MIC6) to their exploitation as diagnostic targets and vaccine candidates [74].

A study delineated the targeting signals required for the microneme protein MIC3 to reach its secretory organelle, revealing that the propeptide, combined with any one of its three EGF domains, is sufficient for correct trafficking. Importantly, elements critical for MIC3′s adhesive function – including propeptide cleavage, dimerization, and the chitin-binding-like domain – are dispensable for targeting, highlighting a separation between protein localization and functional maturation [75]. The research also notes that MIC3 storage is regulated in a cell cycle-dependent manner, adding a temporal layer to the control of microneme protein availability. The dissociation between sorting determinants and adhesive domains in MIC3 [75], and the interchangeable aliphatic propeptide motifs shared by multiple MICs [74], together suggest that the microneme targeting apparatus is highly conserved and generic, whereas functional specialization is encoded in the ectodomain architecture of each protein. This distinction has practical relevance: modifications to surface-exposed functional domains for vaccine or diagnostic purposes need not disturb the trafficking signals, facilitating the rational engineering of recombinant MIC antigens.

## 5. Functional roles of individual microneme proteins

The individual microneme proteins described below differ substantially in their domain composition, host cell ligands, stage-specific expression, and contributions to pathogenesis. A comparative overview of their key properties is provided in Table 1. Although these proteins share the common destination of the microneme organelle and broadly contribute to host cell adhesion and invasion, several lines of evidence – including gene knockout phenotypes, structural studies, and expression profiling – reveal that they are not functionally interchangeable. Understanding these distinctions is essential for prioritizing MIC proteins as diagnostic markers or vaccine candidates since efficacy in one context does not predict utility in another.

**Table 1.** Summary of key *T. gondii* microneme proteins, their molecular functions, and translational relevance.

Protein	Type	Key molecular function	Diagnostic utility	References
MIC1	Soluble	Sialic acid-binding lectin; complex formation with MIC4/MIC6; TLR4 activation; macrophage pyroptosis *via* NLRP3	96.1% sensitivity (acute); combined with MAG1 + MIC3 reaches 88.9% for chronic infection	[55,98,102,105,114]
MIC2	Transmembrane (TRAP-family)	Gliding motility; host cell attachment under shear stress; helical invasion; M2AP trafficking	rMIC2-MIC3 fusion: high sensitivity without cross-reactivity	[63,67,106,117]
MIC3	Soluble	Host cell adhesion; TLR11 activation (murine); EGF-mediated EGFR-Akt inhibition of autophagy; all life cycle stages	Combined with MIC1ex2 + MAG1: 88.9% sensitivity for chronic infection; cross-reactive with N. caninum	[80,81,85,87,105,108,128]
MIC4	Soluble	Galactose-binding lectin; six apple domains; TLR2/TLR4 activation; Th1 priming	T-cell memory assay: IFN-γ induction with 100% sensitivity, 86–100% specificity for chronic infection	[48,56,59,98,130]
MIC6	Transmembrane (Escorter)	Escort for MIC1/MIC4; aldolase-binding; EGFR-Akt autophagy inhibition; virulence determinant in Chinese-1 lineage	IFN-γ induction from CD4+ and CD8+ T cells; 100% sensitivity for chronic infection	[48,58,62,86,114,124]
MIC8	Transmembrane	Moving junction formation; drug resistance-associated regulation; essential invasion factor	pMIC8 peptide: acute-phase specific marker (recognized within first 3 months post-infection)	[88–90,107,125,127]
MIC13	Soluble (Sialic acid-binding)	Upregulated under bradyzoite/stress conditions; essential for proliferation under alkaline/low-O₂ conditions; intestinal dissemination	Limited direct diagnostic data; component of MGS/MGS1 multi-epitope constructs	[56,90–94,125]
MIC17A	Soluble	Highly expressed in merozoite stage; limited tachyzoite expression	Superior marker for feline toxoplasmosis diagnosis; merozoite antigens more reactive with feline antibodies than GRA1 or MIC3	[109]

### 5.1. MIC2: the TRAP-family adhesin

MIC2 is a protein involved in the gliding motility of *T. gondii* and the attachment to the host cell surface [76]. It has been well established that apicomplexan parasites depend on TRAP family adhesins for actin-based motility and host cell invasion [77]. In *T. gondii*, the TRAP ortholog MIC2 possesses a short cytoplasmic tail essential for motility, with aldolase bridging actin filaments and the MIC2 cytoplasmic domain. The MIC2 tail contains a conserved penultimate tryptophan required for aldolase binding, along with clustered acidic residues. Two distinct acidic clusters in the MIC2 cytoplasmic domain are essential for parasite survival. The C-terminal cluster is required for direct aldolase interaction, whereas the upstream cluster, though dispensable for aldolase binding, remains critical for survival. Conservation of both acidic motifs across TRAP orthologs suggests a central role in apicomplexan motility [77].

A notable point of contention in the MIC2 literature concerns the degree to which this protein is essential for invasion. In a functional analysis of MIC2, the *T. gondii* homolog of the conserved TRAP-family adhesin, a study clarifies and refines its role in parasite motility and invasion [67]. While MIC2 is confirmed as critical for efficient host cell surface attachment under shear stress, the data indicate it is not essential for sustaining gliding motility or the core invasion process once initiated, as parasite speed remains unaffected. Notably, deletion of *mic2* resulted in only a mild attenuation of virulence *in vivo* and a delayed egress phenotype *in vitro*, suggesting a modulatory rather than an indispensable role. This challenges the presumption of essentiality for TRAP-family proteins in apicomplexan motility and positions MIC2 primarily as a key facilitator for surface attachment with secondary roles in signaling and egress [67]. This finding stands in apparent conflict with earlier conditional knockdown data demonstrating that suppression of MIC2 severely impairs invasion and abrogates lethality in mice [64]. One possible reconciliation is that complete transcriptional suppression and genetic deletion differ in the residual protein levels they produce; alternatively, compensatory upregulation of other TRAP-family or adhesive proteins may partially rescue the deletion phenotype. Resolving this discrepancy through complementation studies with domain-specific point mutants would clarify whether the cytoplasmic tail or the ectodomain is the critical functional module for *in vivo* virulence.

A study examines the extensive glycosylation of the microneme protein MIC2, a key adhesin in *T. gondii*, identifying specific C-linked and O-linked modification sites added by the enzyme TgPOFUT2 [78]. Contrary to findings in related parasites, this O-glycosylation is dispensable for tachyzoite function, as genetic disruption of TgPOFUT2 resulted in only modest effects on MIC2 levels and no significant impact on parasite invasion or plaque formation, indicating this modification is not essential for MIC2’s role in infectivity [78]. A yeast-two-hybrid study identifies two novel host protein interactors for the microneme adhesin MIC2: LAMTOR1, involved in lysosome maturation and signaling, and RNaseH2B, associated with RNA catabolism [79]. These interactions suggest that MIC2 may function beyond physical adhesion, potentially modulating host cell signaling pathways and other intracellular processes during infection [79]. If confirmed in cell-based assays, such host-pathway manipulation would substantially broaden MIC2’s role from a mechanical adhesin to a multifunctional effector protein with immunomodulatory potential.

### 5.2. MIC3: a multifunctional adhesin and immunomodulator

Among the various proteins implicated in parasite biology, microneme protein 3 (MIC3) stands out as a secreted protein expressed throughout all stages of the *T. gondii* life cycle, demonstrating strong immunoreactivity and playing pivotal roles in host cell recognition, adhesion, and invasion processes [80]. Unlike MIC2, which is expressed predominantly in tachyzoites and shows stage-specific regulation, MIC3′s pan-stage expression makes it particularly attractive as a universal serodiagnostic target capable of detecting both acute and chronic infection phases. Understanding the molecular structure of MIC3, its mechanisms in parasitic invasion, its association with virulence, and its capacity to induce protective immunity provides essential groundwork for developing diagnostic tools and vaccines against toxoplasmosis [80]. MIC3 has all the above-mentioned MIC properties and has been reported to have excellent immune reactivity [81].

Comparative analyses have revealed elevated expression levels of MIC3 in highly pathogenic Toxoplasma strains relative to attenuated variants [82]. Additionally, this protein exhibits a potent capacity to induce M1 macrophage polarization and stimulate tumor necrosis factor alpha (TNF-α) secretion. The clinical relevance of these findings is corroborated by elevated TNF-α levels detected in patients presenting with cerebral or ocular toxoplasmosis, thereby implicating tachyzoite-derived secretory products in driving inflammatory responses [83].

A study significantly expands the functional scope of MIC3 by identifying specific host protein interactors: Spata3 and Dkk2, which are involved in reproduction, growth, and development, with its tandem EGF domains mediating these interactions [84]. This positions MIC3 as a cross-domain signalling molecule that interfaces with host developmental pathways far beyond immediate invasion processes – an observation with potentially underappreciated implications for congenital toxoplasmosis, where disruption of developmental signalling in the placenta or fetus could contribute to adverse outcomes independently of direct cytopathic invasion.

A study identifies a peptide from the *Toxoplasma* microneme protein MIC3 as a novel, species-specific immune activator [85]. In mouse macrophages, the MIC3 peptide triggers a pro-inflammatory response (TNF-α, iNOS, Ly6C) *via* the TLR11/MyD88/NF-κB pathway. However, because human macrophages lack TLR11, MIC3 fails to induce this response, highlighting a key difference in immune recognition between hosts. Importantly, MIC3 is structurally and functionally distinct from the known TLR11 ligand profilin, as it does not induce IL-12 [85]. This species-specific divergence in TLR11 recognition has critical implications for translating murine vaccine and immunotherapy findings to humans: protective mechanisms observed in mouse models that depend on TLR11-driven immunity may not replicate in human clinical settings, and vaccine formulations for human use may require adjuvants that compensate for the absence of TLR11 signalling.

### 5.3. MIC6: escort protein and virulence determinant

MIC6 is the escorter of MIC1 and MIC4 protein [58]. A study identifies a key mechanistic link between microneme-based adhesion and parasite motility, demonstrating that the cytoplasmic domain of MIC6 directly interacts with aldolase, thereby tethering the MIC1/4/6 adhesive complex to the actin cytoskeleton [62]. Notably, MIC6 thus serves dual functions: as a trafficking escort ensuring the MIC1/MIC4 complex reaches the microneme organelle, and as a direct mechanical linker coupling extracellular adhesion to intracellular force generation. This dual role distinguishes MIC6 from soluble adhesins such as MIC1 and MIC4, which depend on MIC6 for both their localization and their mechanical coupling to the motor apparatus.

A study identifies TgMIC6 as a key virulence determinant in the dominant Chinese 1 lineage of *T. gondii* [86]. Using CRISPR/Cas9 to create a MIC6 knockout (WH3-Δmic6), researchers found that the deletion significantly reduced parasite proliferation, cytokine responses, and mortality in mice. The mechanism involves MIC6 enhancing virulence by inhibiting host cell autophagy, specifically through activating the EGFR-Akt-mTOR pathway. Furthermore, MIC6 contributes to immune evasion; when the host immune signal CD40L was blocked, the virulence of the MIC6-knockout strain was partially restored [86]. Research reveals that MIC3 and MIC6, through their EGF-like domains, activate the host’s EGFR-Akt signalling pathway to inhibit autophagy, thereby maintaining the parasitophorous vacuole’s non-fusogenic nature and promoting intracellular survival [87]. The convergence of MIC3 and MIC6 on the same EGFR-Akt axis – despite their distinct structural contexts – suggests that EGF-domain-mediated EGFR activation is a conserved immune evasion strategy shared by multiple MIC family members [87]. This redundancy implies that blocking a single MIC protein may be insufficient to restore autophagic clearance of the parasite. Targeting EGFR signalling downstream of MIC binding, or simultaneously neutralizing both MIC3 and MIC6 EGF domains, may be required for effective therapeutic intervention.

### 5.4. MIC8: essential for moving junction formation

A study identifies MIC8 as an essential invasion factor in *T. gondii*, specifically required for the formation of the moving junction with the host cell [88]. Its unique cytoplasmic domain is critical for this function and cannot be complemented by other micronemal proteins, distinguishing MIC8 as a novel and functionally distinct component of the invasion machinery [88]. This non-redundancy sets MIC8 apart from many other MIC proteins, which exhibit overlapping functional roles; the inability of other MICs to compensate for MIC8 loss suggests that moving junction formation depends on unique structural features of MIC8’s cytoplasmic domain that are not present in other family members.

A study examining monensin resistance in *T. gondii* reveals that MIC8 was downregulated in the resistant strain alongside an upregulation of actin [89]. This proteomic shift suggests an altered invasion mechanism that specifically impairs the formation of moving junctions with host cells, leading to reduced invasion and egress capabilities while enhancing intracellular replication [89]. The fact that drug-resistant parasites downregulate MIC8 to shift from junction-dependent invasion to a replication-focused intracellular strategy highlights an adaptive plasticity in MIC protein usage that has implications for therapeutic resistance: interventions designed around inhibiting moving junction formation may inadvertently select for MIC8-low, replication-enhanced parasite variants.

### 5.5. MIC13: a stress-responsive microneme protein

The MIC family participates in the intricate process through which the parasite penetrates host cells. Within this group, MIC13 appears particularly significant for enabling parasite spread throughout the body by engaging with intestinal epithelial tissue [90]. Unlike most micronemal proteins, which exhibit high abundance during the rapidly replicating tachyzoite stage to support cellular infection, MIC13 shows minimal tachyzoite expression and substantial upregulation in the slowly dividing bradyzoite form or under environmental stress [56]. This distinctive expression profile fundamentally distinguishes MIC13 from adhesins such as MIC2 and MIC3, and has direct implications for diagnostics: because MIC13 is not prominently expressed during the tachyzoite phase responsible for acute infection, it is unlikely to generate strong antibody responses detectable in early-infection serology, limiting its utility as a standalone acute-phase marker.

Consistent with this expression pattern, experimental evidence indicates that MIC13 does not contribute to tachyzoite host cell entry, intracellular multiplication, or cellular exit, nor does it influence acute disease severity in murine models [91]. Successful CRISPR/Cas9 disruption revealed that while MIC13 is dispensable for normal tachyzoite growth and virulence, it is essential for optimal parasite proliferation under stress such as high pH or low oxygen, marking it as the first micronemal protein implicated in adapting to the chronic infection niche [92]. These findings position MIC13 as a functionally specialized MIC uniquely adapted to the bradyzoite and chronic-phase biology of *T. gondii*, rather than a canonical invasion adhesin. Its inclusion in multi-epitope vaccine constructs [93,94], is therefore best justified as a strategy to elicit immunity targeting the chronic parasite reservoir, complementing antigens that target tachyzoite invasion.

### 5.6. MIC16 and other microneme proteins

Building upon the diverse functional and immunological roles of microneme (MIC) proteins, analysis of MIC16 reveals its additional utility as a genetic marker for population studies [95]. While MIC16 is known to function in invasion through aldolase binding and rhomboid cleavage, examination of its sequence across *T. gondii* strains from varied hosts and regions demonstrates limited but consistent genetic variation. Notably, phylogenetic analysis and a developed PCR-RFLP method using these sequences can reliably differentiate the three major clonal lineages (Types I, II, and III), positioning the MIC16 gene as a novel tool for genotyping and epidemiological tracking of isolates [95]. This epidemiological application of MIC16 illustrates how MIC-family proteins can serve purposes well beyond pathogenesis – an expanding functional repertoire that increasingly includes molecular epidemiology, diagnostics, and strain surveillance.

A functional study characterizes key transmembrane microneme proteins (TM-MICs) and reveals their diverse roles in invasion beyond adhesion, demonstrating that while several TM-MICs (including MIC6, MIC8, and AMA1) are cleaved by rhomboid proteases and some bind aldolase to connect to the actin cytoskeleton, their trafficking to micronemes often depends on interactions *via* their ectodomains, not their C-terminal tails [96]. Notably, aldolase binding is essential for AMA1 function but dispensable for MIC6 [96]. Research identifies TgSPATR as a probable new microneme protein, demonstrating through co-localization and secretion studies that it shares key characteristics with established MIC proteins like MIC2 [97]. Its calcium-dependent secretion, surface exposure during invasion, and structural homology to *Plasmodium* SPATR proteins suggest it is likely involved in the early stages of host cell invasion [97].

Comparative analysis across MIC proteins reveals several emerging themes. First, functional redundancy is common for some roles (e.g. gliding motility, sialic acid binding) but absent for others (e.g. moving junction formation by MIC8), meaning that the therapeutic impact of targeting any given MIC protein will depend critically on whether redundant mechanisms can compensate. Second, stage-specific expression (MIC13 in bradyzoites; MIC17A in merozoites) constrains the diagnostic utility of individual proteins but can be exploited to design assays that discriminate infection phase or host species. Third, the expanding list of host interactors for MIC proteins – including EGFR [86,87], LAMTOR1 [79], and developmental regulators [84] – suggests that MIC proteins are increasingly understood as active manipulators of host physiology, not merely structural adhesins. For a consolidated overview of the major microneme proteins discussed above, including their molecular functions and translational potential, see Table 1.

## 6. Immunomodulatory functions of microneme proteins

**Figure 3.**
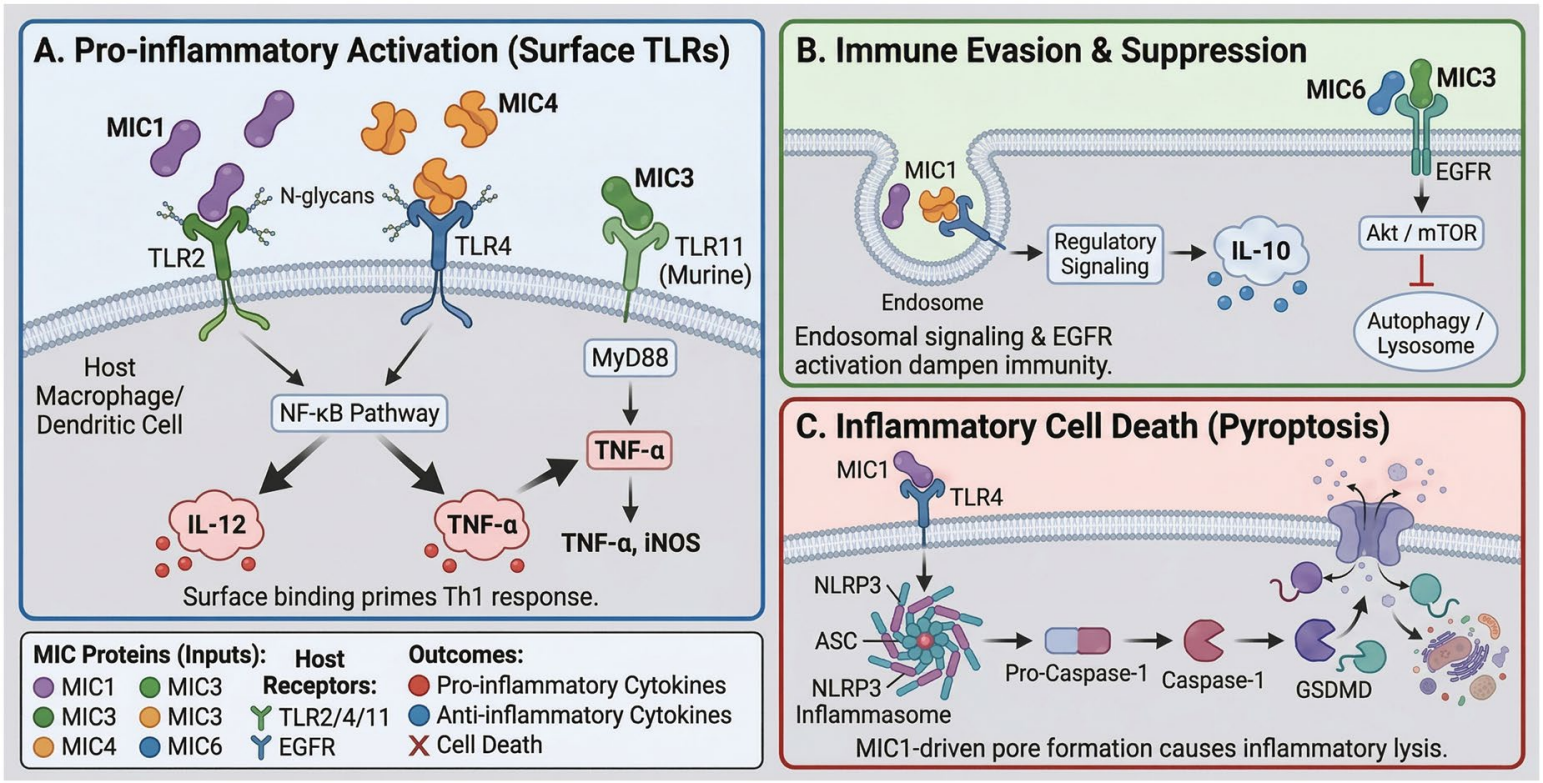
Immunomodulatory functions of *Toxoplasma gondii* microneme proteins. Microneme proteins (MICs) modulate host immune responses through three distinct mechanisms: pro-inflammatory activation, immune evasion/suppression, and inflammatory cell death. (A) Pro-inflammatory Activation *via* Surface TLRs: MIC1 and MIC4 bind to Toll-like receptor 2 (TLR2) and TLR4 on host macrophages and dendritic cells through N-glycan-mediated interactions, activating the NF-κB signaling pathway to induce production of pro-inflammatory cytokines IL-12 and TNF-α, thereby priming a protective Th1 immune response. MIC3 engages TLR11 (in murine cells) through the MyD88-dependent pathway, triggering TNF-α and iNOS expression. This surface TLR binding represents the primary mechanism by which MIC proteins act as pathogen-associated molecular patterns (PAMPs) to alert the host immune system. (B) Immune Evasion and Suppression: Following initial pro-inflammatory activation, MIC proteins employ counter-regulatory mechanisms to dampen host immunity and promote parasite survival. MIC1 bound to TLR4 undergoes endocytosis, triggering endosomal regulatory signaling that induces anti-inflammatory IL-10 production, transiently suppressing the inflammatory response. Simultaneously, MIC3 and MIC6, through their EGF-like domains, activate the host epidermal growth factor receptor (EGFR), which stimulates the Akt/mTOR signaling pathway. This activation inhibits autophagy and prevents lysosomal targeting of the parasitophorous vacuole, thereby maintaining the vacuole’s non-fusogenic nature and ensuring intracellular parasite survival. (C) Inflammatory Cell Death (Pyroptosis): MIC1 can trigger pyroptosis, a form of inflammatory programmed cell death, through TLR4-mediated activation of the NLRP3 inflammasome. Upon MIC1-TLR4 engagement, the NLRP3 inflammasome complex assembles, recruiting the adaptor protein ASC and activating pro-caspase-1 to its active form (caspase-1). Active caspase-1 cleaves gasdermin D (GSDMD), releasing its N-terminal domain that oligomerizes to form pores in the plasma membrane. This MIC1-driven pore formation causes inflammatory lysis of the host cell, releasing pro-inflammatory cytokines and damage-associated molecular patterns.

Beyond their structural roles in adhesion and motility, MIC proteins engage host immune pathways through three principal mechanisms: pro-inflammatory activation *via* surface pattern-recognition receptors, active suppression of immune effector functions, and induction of inflammatory cell death. These mechanisms are not mutually exclusive; several MIC proteins simultaneously activate and suppress immunity, and understanding this duality is critical for designing vaccines that overcome parasite-mediated immune subversion. Beyond their role in adhesion, MIC1 and MIC4 act as immunomodulators (Figure 3) [98,99]. They bind to Toll-like receptors (TLR) 2 and TLR4 on murine antigen-presenting cells, acting as pathogen-associated molecular patterns (PAMPs) to upregulate interleukin (IL)-12 production, thereby priming a protective Th1 immune response [59]. This interaction involves specific N-glycans on TLR2, with MIC1 and MIC4 targeting distinct sites [59]. The immune activation is further modulated by receptor heterodimerization and co-receptor engagement [100], and can also induce the anti-inflammatory cytokine IL-10, suggesting a regulatory role [101].

TgMIC1, a key adhesion protein for *T. gondii* invasion, also regulates host immunity by driving macrophage pyroptosis [102]. A study using a *MIC1* knockout strain (Wh3Δmic1) showed reduced parasite invasion and growth, as well as attenuated inflammatory responses, in mice. Furthermore, recombinant MIC1 (rMIC1) – but not a mutant form (rMIC1-T126A/T220A) lacking TLR4 binding – triggered pyroptosis in macrophages by upregulating NLRP3 and activating caspase-1 and GSDMD. This effect was absent in TLR4- or NLRP3-deficient cells. The study concludes that TgMIC1 plays a dual role in invasion and in modulating host immunity by activating the TLR4/NLRP3 pathway to induce macrophage pyroptosis [102].

A study revealed a dual immunomodulatory function for the *T. gondii* microneme proteins MIC1 and MIC4 [101]. While they bind to surface TLR2/TLR4 to trigger pro-inflammatory cytokine production, such as TNF-α, they simultaneously induce the anti-inflammatory cytokine IL-10. This IL-10 response depends specifically on TLR4 internalization *via* endocytosis. Blocking this internalization prevents IL-10 production without affecting TNF-α release. This mechanism allows the parasite to transiently suppress the host’s inflammatory response, favoring its early survival [101]. This sequential activation-then-suppression dynamic – wherein surface TLR engagement first drives IL-12 and TNF-α production followed by endosomal TLR4 recycling that triggers IL-10 – may represent an evolutionarily optimized strategy that harnesses the host’s own regulatory circuits to moderate anti-parasitic immunity.

MIC3 and MIC6, through their EGF-like domains, activate the host EGFR-Akt signaling pathway to inhibit autophagy, thereby maintaining the parasitophorous vacuole’s non-fusogenic nature and promoting intracellular survival [98]. As noted in Section 5.3, the convergence of two structurally distinct MIC proteins (MIC3 and MIC6) on the same EGFR-Akt axis suggests that autophagy inhibition is a priority virulence strategy for the parasite, with built-in redundancy to ensure effectiveness even if one protein is targeted by host immunity or pharmacological intervention [86,87].

Extending these findings to knockout studies, parasites bearing combined MIC1-3 gene deletions have been shown to elicit potent humoral and Th1-type cellular responses, conferring substantial protection against both chronic infection (exceeding 96% reduction in cerebral cyst burden) and vertical transmission (4.6% fetal infection rate in vaccinated animals versus 33.3% in controls) [103]. The latter finding is especially relevant from a public health perspective: vertical transmission of *T. gondii* during pregnancy is a primary cause of congenital toxoplasmosis, which can result in hydrocephalus, chorioretinitis, and neurodevelopmental impairment in the neonate. The observation that MIC1-3 gene deletions dramatically reduce fetal transmission rates in an experimental model [103] provides a strong rationale for prioritizing MIC-based vaccine strategies in reproductive-age women. However, these data are currently limited to murine models, and translation to human pregnancy would require substantially more evidence, including assessment of vaccine safety in immunocompromised or pregnant individuals.

Collectively, the immunomodulatory properties of MIC proteins reveal a sophisticated duality: many MICs simultaneously activate pro-inflammatory pathways (Th1 priming, pyroptosis) and dampen immunity (IL-10 induction, autophagy inhibition), enabling the parasite to strike a balance between eliciting host defenses sufficient to suppress competing pathogens while avoiding immune-mediated clearance. This immune-balancing act has important implications for vaccine design: adjuvant strategies and antigen combinations should be selected with the explicit aim of overcoming MIC-mediated immune suppression and tilting the host response toward durable protective Th1 immunity rather than the mixed or regulatory responses that natural infection tends to induce. The species-specific limitation of TLR11-driven MIC3 recognition – absent in humans – further highlights the danger of extrapolating murine immunological data to human vaccine development without appropriate mechanistic validation in human cell systems.

## 7. Diagnostic applications of microneme proteins

### 7.1. Overview and rationale for MIC-based serodiagnosis

Accurate serodiagnosis of toxoplasmosis is essential for clinical management, particularly in antenatal screening, among immunocompromised patients, and for veterinary surveillance. In the antenatal context, accurate stage determination is of paramount clinical importance: primary maternal infection during the first trimester carries a low transmission rate (∼15%) but high fetal morbidity if transmission occurs, whereas third-trimester infection results in more frequent transmission (∼65%) but generally milder fetal sequelae. Conventional serological algorithms that rely on IgM positivity and IgG avidity testing have recognized limitations in distinguishing recent seroconversion from persistent low-avidity IgG, creating diagnostic windows that can lead to inappropriate treatment decisions. MIC-based recombinant antigens offer the potential to complement these existing algorithms by providing stage-discriminatory markers – particularly acute-phase-specific antigens such as pMIC8 and high-sensitivity tachyzoite antigens such as the MIC1 N-terminal fragment – that map more precisely onto the serological kinetics of primary infection.

Conventional diagnostic platforms predominantly rely on crude native Toxoplasma lysate antigen (TLA), which, while sensitive, suffers from batch-to-batch variability, risk of incomplete inactivation, high background reactivity, and the inability to discriminate infection stage. Recombinant MIC-based antigens offer significant advantages in this context, including defined composition, standardized production, reduced risk of cross-reactivity, and the potential for stage-specific diagnosis. The following subsections critically evaluate the evidence for individual and combined MIC antigens across diagnostic platforms, with specific attention to sensitivity, specificity, and comparative performance against established standards.

### 7.2. Individual and chimeric MIC antigens: sensitivity and specificity data

Microneme (MIC) proteins, essential for *T. gondii* host cell invasion, have emerged as versatile tools for managing toxoplasmosis, serving both diagnostic and vaccine purposes [104]. In diagnostic applications, recombinant chimeric antigens combining multiple MIC proteins have demonstrated considerable utility. A MIC1-MAG1 fusion protein achieved 90.8% sensitivity in serodiagnosis of human toxoplasmosis – comparable to the 91.8% sensitivity observed with conventional *Toxoplasma* lysate antigen – while notably outperforming multi-antigen combinations and showing particular promise for discriminating between acute and chronic infection phases [105]. Similarly, a novel immunochromatographic test utilizing recombinant rMIC2-MIC3 fusion protein demonstrated high sensitivity without cross-reactivity to related parasites, offering a more economical approach to toxoplasmosis serodiagnosis [106].

Investigations into optimal antigenic regions have identified the second exon-encoded N-terminal fragment of MIC1 (r-MIC1ex2) as particularly valuable, exhibiting 96.1% sensitivity for acute infections, though reduced sensitivity (75%) for chronic infections with low IgG titers [105]. Combining r-MIC1ex2 with MAG1 and MIC3 improved the sensitivity for detecting chronic infection to 88.9%, comparable to established recombinant antigen mixtures and native lysate antigen in ELISA formats [105]. For acute-phase identification, a peptide derived from MIC8 (pMIC8) has been characterized as a specific serological marker, recognized most precisely by antibodies in serum from individuals infected within the first three months, with diminishing recognition up to six months post-infection. Combining pMIC8 with standard soluble antigen effectively distinguishes acute from chronic toxoplasmosis [107]. In the antenatal screening context, this temporal specificity of pMIC8 is particularly valuable: a positive pMIC8 response in a pregnant woman would strongly suggest recent primary infection, enabling timely initiation of antiparasitic chemoprophylaxis and targeted fetal monitoring.

Comparative analysis of these studies reveals several important patterns. First, no single MIC antigen achieves optimal sensitivity across all infection phases: MIC1 fragments excel in acute-phase detection but underperform in chronic low-titer infection, whereas combination antigens are consistently superior for stage-independent screening. Second, chimeric constructs (e.g. rMIC2-MIC3, MIC1-MAG1) reproducibly outperform individual antigens, supporting a multi-epitope approach to antigen design. Third, the sensitivity and specificity reported in individual studies should be interpreted cautiously, as differences in patient cohort composition – particularly the proportion of acute versus chronic cases and the prevalence of immunosuppression – significantly influence observed diagnostic performance. Direct head-to-head comparisons under standardized conditions are still lacking in the literature, representing an important unmet need. Fourth, virtually all validation studies have been conducted in single-centre settings without external validation cohorts, which limits generalizability. Direct head-to-head comparisons of MIC-based assays under standardized conditions are still lacking in the literature, representing an important unmet need that must be addressed before any MIC-based platform can be recommended for widespread clinical adoption.

The differential expression of MIC antigens across parasite life-cycle stages (tachyzoite, bradyzoite, merozoite, sporozoite) has direct implications for antenatal diagnostics, where distinguishing primary maternal infection from reactivation is critical for assessing fetal risk. Current serological algorithms rely primarily on IgM and IgG avidity testing; the incorporation of stage-specific MIC antigens – particularly acute-phase markers such as pMIC8 and high-avidity chronic-phase antigens – into next-generation panels could substantially improve the clinical accuracy of gestational timing of infection, thereby guiding antiparasitic treatment decisions.

### 7.4. Cross-reactivity and limitations in MIC-based serodiagnosis

A critical consideration in evaluating MIC-based diagnostic antigens is the potential for cross-reactivity with related apicomplexan parasites. *Toxoplasma gondii* shares genus-level and family-level phylogenetic relationships with *Neospora caninum, Hammondia hammondii, Sarcocystis* species, and *Eimeria* species – all of which are prevalent in livestock populations. MIC3 has been shown to be antigenically cross-reactive with N. caninum [108], which is a significant confounding factor in veterinary serodiagnostic settings where both parasites co-circulate in cattle. Conversely, the rMIC2-MIC3 immunochromatographic assay demonstrated the absence of cross-reactivity with related parasites in the study conditions reported [106]; however, the panel of parasites tested was limited, and systematic cross-reactivity profiling against the full range of relevant co-endemic species has not been reported for most MIC-based assays.

Additional limitations include: (i) the predominant reliance on IgG-based detection, which cannot reliably discriminate recent seroconversion from long-standing chronic infection without IgM or avidity co-testing; (ii) variability in the sensitivity of MIC antigens across different *T. gondii* genotypes (Types I, II, and III have different virulence profiles and may elicit quantitatively distinct antibody responses to specific MIC epitopes); and (iii) the absence of large, multi-centre, prospectively validated studies for most MIC-based assays, which limits their regulatory approval and widespread clinical adoption. Furthermore, because MIC protein expression levels vary with parasite genotype and infection stage, MIC-based seroassays may perform differently across geographic regions with distinct dominant *T. gondii* strains – a variable that has not been systematically investigated. Addressing these gaps through standardized multi-site validation studies represents a priority for the field.

### 7.5. Stage-specific and host-specific diagnostic considerations

Host-specific diagnostic considerations have revealed that MIC17A, highly expressed during the merozoite stage, is a superior marker for detecting toxoplasmosis in cats. Merozoite-stage antigens react more effectively with feline antibodies than tachyzoite or bradyzoite antigens such as GRA1 and MIC3, indicating that merozoite-specific proteins are optimal for reliable serological diagnosis in the definitive host – a critical factor for controlling transmission [109]. This highlights a broader principle: the optimal diagnostic antigen is host-specific and stage-specific, and assays validated in one host (e.g. mice or humans) cannot be assumed to perform equivalently in the definitive feline host or in livestock intermediate hosts.

Beyond humoral responses, MIC proteins demonstrate a significant capacity to stimulate cellular immunity. MIC1, MIC3, MIC4, and MIC6 are potent inducers of T-cell memory, stimulating IFN-γ production from both CD4+ and CD8+ T cells with 100% sensitivity and 86–100% specificity for detecting chronic infection [48]. The responding T cells are predominantly of the effector memory phenotype, and broad recognition across mouse strains indicates these proteins contain widely presented epitopes, further supporting their diagnostic and immunological significance [48,104]. T-cell-based assays using MIC antigens may offer an important complementary tool to antibody-based serology in immunocompromised patients – such as HIV-positive individuals or organ transplant recipients – in whom humoral responses may be diminished or absent. In this population, in whom toxoplasmic encephalitis from reactivated chronic infection represents a life-threatening complication, cellular immunity assays using MIC4 and MIC6 epitopes may provide diagnostic information that serology cannot, though this application remains to be formally validated.

## 8. Vaccine development strategies employing microneme proteins

Protective immune responses against *T. gondii* can be elicited by several antigenic protein families, including dense-granule proteins (GRAs), surface antigens (SAGs), micronemal proteins (MICs), and rhoptry proteins (ROPs) [110,111]. Much pathogenic and immunogenic evidence has established that MIC1, MIC3, MIC4, and MIC6 serve critical functions in parasite pathogenicity, whereas MIC3, MIC4, MIC5, MIC6, MIC8, and MIC13 have been characterized as highly immunogenic proteins [112,113]. This immunostimulatory capacity underpins their potential as vaccine candidates, as immunization with these proteins confers protective immunity in mice [98,114]. It must be emphasized, however, that the entirety of preclinical vaccine data discussed below derives from murine models – predominantly inbred BALB/c and C57BL/6 strains challenged with laboratory-adapted parasite strains. The translation of murine efficacy data to human populations is complicated by differences in MHC diversity, TLR11 signaling (absent in humans), variability in parasite strains, and the absence of any completed Phase I or Phase II human clinical trials for any MIC-based vaccine. These limitations should be borne in mind when evaluating claims of protective efficacy throughout this section.

### 8.1. Recombinant subunit and protein vaccines

A study advances the development of MIC-based subunit vaccines by demonstrating that a protein-protein vaccine combining the chimeric antigen rEC2 (containing MIC2 and MIC3 fragments) with rGRA7 induces a potent Th1-focused immune response and confers strong protection (79%) against chronic brain cyst formation in mice [115]. In contrast, a heterologous protein-DNA vaccine elicited a mixed Th1/Th2 response and was less protective. The findings underscore that microneme proteins (MIC2 and MIC3) are valuable protective antigens and highlight the critical importance of vaccine formulation and adjuvant choice in achieving effective, Th1-driven immunity against toxoplasmosis [115].

Host cell invasion by *T. gondii* requires apical release of micronemal proteins (MICs) [98]. The protective effect of immunization with MIC1 and MIC4 was evaluated in C57BL/6 mice. Immunized mice developed high levels of MIC1/4-specific IgG1 and IgG2b antibodies and mounted a polarized Th1 response characterized by IL-2, IL-12, IFN-γ, and IL-10 production. Following oral challenge with ME49 cysts, immunized mice exhibited 68% fewer brain cysts and 80% survival compared to 100% mortality in controls. Immunized mice also showed reduced parasitism in lungs and intestine without signs of intestinal necrosis. These findings demonstrate that MIC1 and MIC4 elicit protective immunity against toxoplasmosis, supporting their potential as vaccine candidates [98].

Furthermore, MIC16 has demonstrated promise as a subunit vaccine, eliciting protective immune responses and increasing survival in challenged mice, collectively highlighting the immunogenic value of MIC proteins for diagnosis and prevention [104]. A study identifies synthetic peptides derived from microneme (MIC) proteins as promising vaccine targets [116], demonstrating that immunization with MIC peptides in mice induces potent IgG antibodies and critically shifts the immune response toward a protective Th1 profile with high IFN-γ/IL-10 ratios, which correlates with a significantly reduced parasite burden in the brain during chronic infection, highlighting the vaccine potential of MIC components [116].

While these subunit vaccine studies consistently demonstrate Th1 induction and statistically significant reductions in brain cyst burden, the degree of protection is variable (ranging from 40% to 79% across studies) and is generally assessed relative to naïve unvaccinated controls rather than to established veterinary vaccines such as Toxovax^®^. Future studies should include head-to-head comparisons with licensed vaccines, and should report standardized outcome measures including cyst burden, seroconversion rates, and survival across multiple parasite challenge doses.

### 8.2. DNA vaccines

DNA vaccine platforms have been extensively explored for MIC antigens, with variable efficacy across individual proteins. A study reported that DNA vaccines encoding MIC2 could achieve 40% and 37.5% survival in immunized BALB/c and C57BL/6 mice, respectively, *via* gene gun immunization [117]. The protection provided by MIC3 DNA vaccines is less stable than that of the other antigens [118–121]. MIC6 DNA vaccines could provide adequate protection in mice against acute infection. The highest reported survival rate was 40%, while the remaining results showed a survival extension of 10–20 days [114]. Moreover, when co-administered with the MIC1 and MIC4 cocktail, the vaccine’s protection can be further enhanced, with 75% of mice surviving the acute challenge and a 68% reduction in brain cysts in chronic infection [114]. Other cocktails made from MIC6 can also prolong survival length by 29 days and reduce brain cysts by 65% and 80% [122,123].

Research demonstrates that a DNA vaccine encoding MIC3 (pcDNA3-MIC3) is a potent immunogen, significantly prolonging survival in mice challenged with virulent *T. gondii* [81]. The vaccine elicited strong humoral and cellular immunity, characterized by a specific increase in CD8+ T lymphocytes, indicating a protective role for CD8+ cytotoxic T-cells (CTL). These results solidify MIC3 as a promising vaccine candidate against toxoplasmosis [81].

A study demonstrates that a DNA vaccine expressing microneme protein 6 (pVAX-MIC6) induces a potent mixed Th1-biased immune response – characterized by specific antibodies, T-cell proliferation, and elevated cytokines (IFN-γ, IL-2, IL-4, IL-10) – in mice [124]. Immunization significantly prolonged survival after a lethal challenge, establishing MIC6 as a promising subunit vaccine candidate against toxoplasmosis [124].

MIC8, MIC11, and MIC13 are all proteins involved in adhesion. DNA vaccines based on MIC8 and MIC13 resulted in prolonged survival of 6–27 days in acute challenge and a 25–64% reduction in cysts in chronic challenge [90,125]. Meanwhile, MIC11 vaccines achieved 17% protection against acute infections [126]. The MIC8 DNA vaccine induces strong humoral and cellular immune responses and produces cytokines such as IL-15 and IL-21, which were protective against *T. gondii* challenge [125,127]. Regarding combination vaccine approaches, DNA vaccines encoding MIC5 and MIC16 have demonstrated protective efficacy by inducing elevated IgG titers, enhanced cytokine responses including IFN-γ, IL-2, IL-12p70, and IL-12p40, as well as expanded CD4+ and CD8+ T-cell populations. Correspondingly, vaccinated mice exhibited prolonged survival and reduced cerebral cyst formation compared to unvaccinated controls [40]. Notably, the superior outcomes observed with this bivalent MIC5/MIC16 formulation relative to monovalent preparations provide compelling support for combinatorial approaches in microneme-based vaccine design against toxoplasmosis. Building upon these observations, recent vaccine development efforts have utilized computational epitope prediction to design a multi-epitope construct designated MRS, incorporating immunogenic sequences from MIC3, ROP8, and SAG1. Immunization of BALB/c mice with this construct elicited robust humoral responses and Th1-biased cellular immunity, thereby supporting multi-epitope vaccination as a viable protective strategy [128]. In a complementary approach, studies employing MIC3-encoding DNA vaccines co-administered with IL-12 have similarly demonstrated enhanced Th1 polarization characterized by elevated interferon-gamma production [129].

A cross-cutting limitation of DNA vaccine studies is the consistently modest absolute protection observed in lethal acute challenge models (typically 37–75% survival), which falls substantially short of the near-complete protection required for a clinically deployable human vaccine. This performance gap may reflect the inherent limitations of DNA vaccination platforms – including inefficient antigen expression in non-human primates and humans – and suggests that protein-based or viral-vectored delivery systems may be required to achieve the immunogenicity necessary for clinical translation.

### 8.3. Multi-epitope chimeric vaccines

Multi-epitope chimeric vaccine design represents a rational strategy for combining the immunogenic advantages of individual MIC proteins into a single recombinant construct, while potentially overcoming the insufficient protection observed with monovalent approaches. A study found a multi-epitope vaccine (RMS) against *T. gondii* by combining immunogenic fragments from three key antigens: ROP18, MIC4, and SAG1 [130]. In addition, they proved that the MIC4-containing chimeric protein is a promising vaccine candidate against toxoplasmosis [130].

A study evaluated a novel multi-epitope vaccine candidate against toxoplasmosis in mice [93]. The vaccine consists of a single recombinant protein, MGS, which is a chimera containing epitopes from three *T. gondii* antigens (MIC13, GRA1, SAG1). Administered alone or with adjuvants, it significantly boosted protective immune responses, particularly with Freund’s adjuvant, and improved mouse survival after parasite challenge. The results support the multi-epitope strategy as promising for vaccine development [93].

Consistently, a study used an in silico approach to design a recombinant multi-antigen vaccine (MGS1) against *T. gondii* [94]. A key component is MIC13, a protein critical for parasite dissemination within the host. A selected immunogenic fragment of MIC13 (amino acids 73–272) was fused with fragments from GRA1 and SAG1 using a linker. The resulting 59.56 kDa MGS1 protein showed good, predicted antigenicity (0.6340) and was successfully cloned, expressed, and confirmed in *E. coli*. The work highlights MIC13 as a strategic target to induce immunity against the parasite’s spread, positioning MGS1 as a promising vaccine candidate for future animal model testing [94]. Although computational analyses have identified multiple promising B-cell and T-cell epitopes within MIC4 [131], experimental validation remains necessary to definitively establish its candidacy for vaccine development.

The multi-epitope constructs described above have been evaluated only in initial mouse immunogenicity studies or, in the case of MGS1, only at the stage of recombinant protein production. None have undergone challenge studies in larger animal models or clinical evaluation. The adjuvant dependence observed for the MGS construct – with significantly better outcomes using Freund’s adjuvant, which is not clinically acceptable for human use – illustrates a translational barrier that must be resolved before multi-epitope MIC vaccines can advance to clinical trials.

### 8.4. Live-Attenuated vaccine strains

Low-virulence RH strains with MIC1 and MIC3 gene knockouts have been shown to protect mice against *T. gondii* cyst challenge [132]. Beyond diagnostic applications, the MIC2 protein complex has been identified as a primary virulence determinant in *Toxoplasma* infection. This transmembrane adhesin, in conjunction with its binding partner M2AP, facilitates a principal invasion pathway. Consequently, genetic ablation or reduced expression of MIC2 results in aberrant M2AP trafficking, thereby compromising helical gliding motility, host cell attachment, and cellular invasion. These cumulative defects ultimately abolish lethal infection capacity in murine acute toxoplasmosis models. Of particular significance, MIC2-deficient parasites have demonstrated efficacy as live-attenuated vaccine candidates, conferring improved survival rates, reduced parasite loads, attenuated inflammatory responses, and durable protective immunity [63].

A study used a conditional gene knockdown system to definitively prove that the MIC2-M2AP protein complex is essential for *T. gondii* virulence [63]. By suppressing MIC2 expression, the researchers showed that it is required for host cell attachment, invasion, and the parasite’s unique helical gliding motility. In a mouse model, MIC2-deficient parasites were unable to cause acute lethal infection. Notably, this attenuation led to a lower parasite burden, a reduced inflammatory response, and, crucially, the induction of long-term protective immunity. The findings establish the MIC2 complex as a major virulence factor and demonstrate that MIC2-deficient parasites function as an effective live-attenuated vaccine [63].

A study demonstrates that a live-attenuated *T. gondii* strain with deletions in the microneme protein genes *MIC1* and *MIC3* (MIC1-3KO) is a highly effective vaccine candidate against toxoplasmosis in sheep [133]. Vaccination induced a strong, persistent IgG response and, critically, protected 62–91% of pregnancies from abortion following a mid-gestation challenge, matching the efficacy of the commercial vaccine Toxovax^®^. The mutant strain formed non-infectious tissue cysts and was effective at a lower dose and *via* different administration routes, highlighting its practical potential. This underscores the value of targeting microneme proteins (MIC1 and MIC3) for the development of safe and potent live veterinary vaccines [133]. The performance of MIC1-3KO comparable to Toxovax^®^ in sheep is arguably the strongest translational evidence for MIC-based vaccines reported to date, as it involves an outbred natural host species under conditions relevant to agricultural practice. Nevertheless, several caveats apply: safety studies in immunocompromised animals have not been widely reported; the risk of reversion to virulence requires formal assessment; and regulatory pathways for live genetically modified organisms in food animals vary substantially across jurisdictions. For human vaccine applications, live-attenuated parasite vaccines raise additional safety concerns that have thus far precluded their development, and this remains a principal challenge for the field.

### 8.5. Cross-protective vaccines against related parasites

Cross-protective vaccines targeting conserved antigens shared among related parasites represent an economically and strategically attractive approach. A study identifies MIC3 as a cross-reactive and cross-protective antigen shared between *T. gondii* and the related parasite *Neospora caninum* [108]. Immunization with a TgMIC3-based DNA vaccine (pcDNA3.1-TgMIC3) elicited high antibody titers in mice and provided partial protection against both parasites, prolonging survival after *T. gondii* challenge and reducing brain parasite burden following N. caninum infection [108].

The partial – rather than complete – cross-protection achieved by the TgMIC3 construct indicates that, while antigenic cross-reactivity exists at the antibody recognition level, cross-neutralising functional immunity is incomplete, possibly because critical epitopes differ between the two parasite species at the level of receptor-binding domains. Formulations incorporating species-specific epitopes alongside cross-reactive regions may be required to achieve robust bi-directional protection.

In general, DNA vaccines derived from the MIC protein family demonstrate strong immunogenicity, with types 1, 2, 3, 4, 6, and 11 individually conferring lifelong protection against acute infection. Moreover, MIC-based cocktail vaccines have shown excellent efficacy in both acute and chronic challenge trials, with MIC6 exhibiting particular promise for multi-gene DNA vaccine formulations [134,135]. These proteins, which are exposed on the tachyzoite surface to bind host cell receptors and promote invasion, are key targets; evaluated antigens like MIC2, MIC3, MIC4, MIC8, MIC11, and MIC13 effectively induce robust humoral and Th1-type immune responses, significantly enhancing IFN-γ, IL-12, and IL-2 production and extending survival time [134,135]. The practical utility of these targets is exemplified by the successful recombinant expression of MIC3 from a local *T. gondii* isolate in *E. coli*, a step toward its development as a diagnostic tool or vaccine candidate [136]. Significantly, the function of MICs extends beyond invasion to include facilitation of parasite motility, intracellular survival, and egress, processes governed by their ectodomains, transmembrane regions, and cytoplasmic tails [137]. Given this central role in pathogenesis, MICs are considered promising targets for novel interventions [137], a perspective reinforced by studies of essential complexes like TgMIC1/4/6, whose understanding provides a foundation for developing new control strategies against toxoplasmosis [40]. Across vaccine modalities, several overarching observations emerge. First, cocktail or multi-epitope formulations consistently outperform monovalent preparations, supporting the inclusion of multiple MIC antigens in next-generation vaccine designs. Second, Th1-biased responses – characterized by IFN-γ and IL-12 production – correlate most consistently with protection across studies, whereas mixed Th1/Th2 responses are less protective. Third, no MIC-based vaccine candidate has yet advanced to human clinical trials, and the field remains entirely dependent on murine and, to a lesser extent, ovine or bovine preclinical data. Bridging this translational gap will require investment in non-human primate studies, standardized challenge models with clinically relevant parasite strains, and the development of clinically acceptable adjuvant systems that can replicate the Th1-polarizing effect of Freund’s adjuvant without its inflammatory toxicity. For ease of reference, a summary of various microneme protein-based vaccine strategies against *T. gondii is* provided in Table 2.

**Table 2.** Summary of microneme protein-based vaccine strategies against *T. gondii.*

Vaccine type	Antigen(s)	Formulation/Platform	Immune response	Protection outcome	Model	Reference
Recombinant subunit	rEC2 (MIC2/MIC3) + rGRA7	Protein-protein	Potent Th1-focused response	79% reduction in brain cysts	Mice	[115]
Recombinant subunit	MIC1 + MIC4	Protein immunization	High IgG1/IgG2b; Th1 (IL-2, IL-12, IFN-γ, IL-10)	68% fewer brain cysts; 80% survival	C57BL/6 mice	[98]
Recombinant subunit	MIC16	Subunit vaccine	Protective immune response	Increased survival	Mice	[104]
Recombinant subunit	MIC-derived peptides	Synthetic peptides	Strong IgG; Th1 with high IFN-γ/IL-10 ratio	Reduced brain parasite burden	Mice	[116]
DNA (Single)	MIC2	Gene gun delivery	Humoral and cellular immunity	40% survival (BALB/c); 37.5% (C57BL/6)	Mice	[117]
DNA (Single)	MIC3	pcDNA3-MIC3	Strong humoral/cellular; increased CD8+ T cells	Significantly prolonged survival	Mice	[81]
DNA (Single)	MIC3	DNA vaccine	Variable immune response	Less stable protection than other MICs	Mice	[118–121]
DNA (Single)	MIC6	pVAX-MIC6	Mixed Th1-biased; IFN-γ, IL-2, IL-4, IL-10	Up to 40% survival; 10–20 days extension	Mice	[114,124]
DNA (Single)	MIC8	DNA vaccine	Strong humoral/cellular; IL-15, IL-21	6–27 days prolonged survival; 25–64% cyst reduction	Mice	[90,125,127]
DNA (Single)	MIC11	DNA vaccine	Immune response induction	17% protection against acute infection	Mice	[126]
DNA (Single)	MIC13	DNA vaccine	Humoral and cellular immunity	6–27 days prolonged survival; 25–64% cyst reduction	Mice	[90,125]
DNA (Cocktail)	MIC1 + MIC4 + MIC6	Cocktail DNA	Enhanced Th1 response	75% survival (acute); 68% cyst reduction (chronic)	Mice	[114]
DNA (Cocktail)	MIC6 cocktails	Cocktail DNA	Enhanced immune activation	29 days survival extension; 65–80% cyst reduction	Mice	[122,123]
DNA (Cocktail)	MIC5 + MIC16	Bivalent DNA	Elevated IgG; IFN-γ, IL-2, IL-12; expanded CD4+/CD8+	Prolonged survival; reduced cerebral cysts	Mice	[138]
DNA (Cocktail)	MIC3 + IL-12	DNA with cytokine	Enhanced Th1; elevated IFN-γ	Improved protection	Mice	[129]
Multi-epitope	MRS (MIC3, ROP8, SAG1)	Chimeric construct	Robust humoral; Th1-biased cellular	Protective efficacy demonstrated	BALB/c mice	[128]
Multi-epitope	RMS (ROP18, MIC4, SAG1)	Chimeric construct	Promising immunogenicity	Identified as promising candidate	—	[130]
Multi-epitope	MGS (MIC13, GRA1, SAG1)	Recombinant chimera	Boosted responses (with Freund’s adjuvant)	Improved survival post-challenge	Mice	[93]
Multi-epitope	MGS1 (MIC13, GRA1, SAG1)	Recombinant chimera	Predicted antigenicity: 0.6340	Awaiting animal testing	In silico/E. coli	[94]
Multi-epitope	MIC4 epitopes	Computational analysis	Multiple B-cell and T-cell epitopes identified	Awaiting experimental validation	In silico	[131]
Live-attenuated	MIC1-3KO strain	Gene knockout	Strong, persistent IgG response	62–91% pregnancy protection; matched Toxovax^®^	Sheep	[133]
Live-attenuated	MIC1/MIC3 knockout (RH)	Gene knockout	Reduced virulence	Protection against cyst challenge	Mice	[132]
Live-attenuated	MIC2-deficient parasites	Conditional knockdown	Durable protective immunity	Improved survival; reduced parasite load; attenuated inflammation	Mice	[64]
Cross-protective	TgMIC3	pcDNA3.1-TgMIC3	High antibody titers	Prolonged survival (*T. gondii*); reduced brain burden (*N. caninum*)	Mice	[108]
Recombinant Protein	MIC3	pET-32a(+) expression	Confirmed by SDS-PAGE and immunoblotting	Potential diagnostic/vaccine candidate	*E. coli*	[96,136]

## 9. Current limitations and challenges

Despite the substantial progress documented in preceding sections, it is essential to critically acknowledge the limitations of current evidence and the considerable challenges that must be overcome before MIC-based interventions can achieve routine clinical or veterinary implementation. These limitations span both the diagnostic and vaccine domains and reflect gaps in experimental design, biological understanding, and translational validation.

On the diagnostic side, antigenic cross-reactivity remains a substantive and unresolved challenge, particularly in veterinary settings, where MIC3 has been shown to cross-react with *Neospora caninum*. Although chimeric antigen design partially mitigates this issue, systematic cross-reactivity profiling against the full spectrum of co-endemic apicomplexans is lacking for most validated MIC antigens. Compounding this problem is the stage-specific nature of MIC protein expression. Because MIC proteins are differentially expressed across tachyzoite, bradyzoite, and merozoite stages, stage-mismatched antigens risk producing false-negative results in specific clinical contexts. MIC13, for instance, is predominantly upregulated in bradyzoites, which calls into question its utility as a diagnostic antigen for the tachyzoite phase and underscores the need for validation across diverse patient populations. Beyond these biological constraints, the evidence base itself remains structurally limited. Most of the sensitivity and specificity data for MIC-based assays originate from single-center studies with relatively small, selected cohorts, and multi-center prospective validation across diverse geographic and clinical settings has not yet been conducted – a prerequisite for regulatory approval and routine use recommendations. Additionally, the extent to which MIC antigen sequence polymorphisms across at least three major clonal lineages of *T. gondii*, as well as numerous atypical strains with distinct virulence profiles, affect serodiagnostic performance remains largely uncharacterized, representing a significant gap in genotype-dependent antigenicity profiling.

The limitations confronting MIC-based vaccine research are equally significant. With the notable exception of the MIC1-3KO sheep trial, virtually all vaccine efficacy data are derived from rodent models, and given that rodent immune responses and infection kinetics differ substantially from those in humans, direct extrapolation of murine protection data to human vaccine efficacy is not warranted. No MIC-based vaccine candidate has entered human Phase I clinical trials to date. Interpretation of the available pre-clinical data is further complicated by considerable variability in reported protection rates, even for the same antigen across different studies. This heterogeneity arises from differences in mouse strain, parasite challenge dose and route, infection stage, adjuvant formulation, and immunization schedule, making direct comparisons and meta-analytic synthesis difficult. A particularly important limitation is that even the most protective MIC-based vaccine strategies reduce, but do not eliminate, brain cyst formation, with most approaches reporting cyst-burden reductions of 60–80%. Complete sterile immunity has not been demonstrated for any MIC-based approach, which carries meaningful implications for the risk of reactivation in immunocompromised individuals. Underlying these practical shortcomings are deeper immunological and biological constraints: the intracellular localization of the parasite, its capacity to modulate host immunity – including through MIC-mediated EGFR-Akt autophagy inhibition – and strain-level variation in MIC expression collectively constitute biological obstacles to durable vaccine-induced immunity. Designing vaccine platforms capable of overcoming these immune-evasion mechanisms, therefore, remains an important and unresolved research priority.

## 10. Conclusion and future perspectives

Microneme proteins represent a functionally diverse and therapeutically significant family of molecules that orchestrate multiple critical processes in *T. gondii* pathogenesis. This comprehensive review has systematically examined the structural organization, regulatory mechanisms, and multifaceted functions of MICs, revealing their central roles not only in the classical invasion paradigm but also in immunomodulation, host cell signaling manipulation, and parasite survival strategies.

The molecular characterization of key MIC complexes – particularly the soluble MIC1/4/6 adhesin complex and the transmembrane MIC2-M2AP complex – has elucidated the sophisticated mechanisms by which *T. gondii* recognizes and invades host cells through lectin-mediated glycan interactions and coordinated actin-myosin motor coupling. The identification of calcium-dependent and PKG-mediated signaling pathways controlling microneme secretion, coupled with the essential role of rhomboid proteases (particularly ROM4) in adhesin processing and turnover, has revealed the intricate regulatory networks governing MIC deployment during invasion.

Beyond their canonical roles in invasion, MICs have emerged as potent immunomodulators that engage host pattern recognition receptors (TLR2, TLR4, and TLR11) to trigger both pro-inflammatory (IL-12, TNF-α) and anti-inflammatory (IL-10) responses. This dual functionality enables the parasite to manipulate host immunity during the early stages of infection. The discovery that specific MICs (MIC3 and MIC6) activate the EGFR-Akt pathway to inhibit autophagy further underscores the sophisticated immune evasion strategies employed by *T. gondii*.

The translational applications of MICs are substantial and continue to expand. In diagnostics, MIC-based antigens (MIC1, MIC3, MIC8, MIC17A) have demonstrated excellent sensitivity and specificity for serodiagnosis, with utility in differentiating acute from chronic infections. The development of chimeric fusion proteins (rMIC2-MIC3, MIC1-MAG1) and stage-specific antigens offers promising avenues for improved diagnostic accuracy. In vaccine development, multiple platforms – including DNA vaccines, recombinant subunit vaccines, multi-epitope chimeric constructs, and live-attenuated strains (MIC1-3KO) – have demonstrated protective efficacy in experimental models, with some approaches (notably the sheep vaccine trial) approaching commercial viability.

However, a balanced assessment of the field requires explicit acknowledgment of current limitations. The diagnostic evidence base for MIC-based antigens remains predominantly derived from single-centre studies with limited prospective multi-site validation, and issues of cross-reactivity with related apicomplexan parasites have not been systematically resolved. For vaccine development, the almost exclusive reliance on rodent efficacy data – combined with the absence of any MIC-based candidate in human clinical trials – means that the clinical translatability of these approaches remains uncertain. Strain-level variation in MIC expression and antigenicity, immune-evasion mechanisms mediated by MIC proteins themselves, and the challenge of achieving sterile immunity are additional obstacles that must be addressed in future research programmes.

Future research should prioritize several key areas: (1) comprehensive structural elucidation of MIC complexes and their interactions with host receptors to enable rational drug design; (2) validation of promising vaccine candidates in relevant animal models and eventual human clinical trials; (3) development of standardized MIC-based diagnostic panels for global implementation; (4) exploration of MIC proteins as targets for novel therapeutic interventions, including small molecule inhibitors of MIC-receptor interactions; and (5) investigation of strain-specific variations in MIC expression and their implications for virulence and vaccine cross-protection.

In conclusion, microneme proteins occupy a central position in *T. gondii* biology, functioning as molecular bridges between parasite and host that determine invasion success, immune response modulation, and disease outcome. The accumulated knowledge regarding MIC structure, function, and immunogenicity provides a robust foundation for developing next-generation diagnostic tools and therapeutic interventions against toxoplasmosis. Continued investment in MIC research promises to yield significant advances in our ability to prevent and treat this globally prevalent parasitic disease, ultimately improving outcomes for vulnerable populations, including immunocompromised individuals, pregnant women, and their offspring.

## Data Availability

No new data were created or analyzed in this study.
